# Decadal Changes in Zooplankton of the Northeast U.S. Continental Shelf

**DOI:** 10.1371/journal.pone.0087720

**Published:** 2014-01-31

**Authors:** Hongsheng Bi, Rubao Ji, Hui Liu, Young-Heon Jo, Jonathan A. Hare

**Affiliations:** 1 Chesapeake Biological Laboratory, University of Maryland Center for Environmental Science, Solomons, Maryland, United States of America; 2 Biology Department, Woods Hole Oceanographic Institution, Woods Hole, Massachusetts, United States of America; 3 Department of Marine Biology, Texas A & M University, Galveston, Texas, United States of America; 4 Department of Oceanography, Pusan National University, Busan, South Korea; 5 Northeast Fisheries Science Center Narragansett Laboratory, National Marine Fisheries Service, Narragansett, Rhode Island, United States of America; University of Connecticut, United States of America

## Abstract

The abundance of the subarctic copepod, *Calanus finmarchicus*, and temperate, shelf copepod, *Centropages typicus*, was estimated from samples collected bi-monthly over the Northeast U.S. continental shelf (NEUS) from 1977–2010. Latitudinal variation in long term trends and seasonal patterns for the two copepod species were examined for four sub-regions: the Gulf of Maine (GOM), Georges Bank (GB), Southern New England (SNE), and Mid-Atlantic Bight (MAB). Results suggested that there was significant difference in long term variation between northern region (GOM and GB), and the MAB for both species. *C. finmarchicus* generally peaked in May – June throughout the entire study region and *Cen. typicus* had a more complex seasonal pattern. Time series analysis revealed that the peak time for *Cen. typicus* switched from November – December to January - March after 1985 in the MAB. The long term abundance of *C. finmarchicus* showed more fluctuation in the MAB than the GOM and GB, whereas the long term abundance of *Cen. typicus* was more variable in the GB than other sub-regions. Alongshore transport was significantly correlated with the abundance of *C. finmarchicus*, i.e., more water from north, higher abundance for *C. finmarchicus*. The abundance of *Cen. typicus* showed positive relationship with the Gulf Stream north wall index (GSNWI) in the GOM and GB, but the GSNWI only explained 12–15% of variation in *Cen. typicus* abundance. In general, the alongshore current was negatively correlated with the GSNWI, suggesting that *Cen. typicus* is more abundant when advection from the north is less. However, the relationship between *Cen. typicus* and alongshore transport was not significant. The present study highlights the importance of spatial scales in the study of marine populations: observed long term changes in the northern region were different from the south for both species.

## Introduction

Zooplankton are drifters in the ocean and often respond to environmental changes rapidly. Zooplankton also play an important role in ecosystems because they link primary producers to planktivorous fish and the intermediary position of zooplankton underscores their significance for food web structure. Rapid changes in zooplankton can potentially have major and rapid effects on higher trophic level species [1]–[2].

In the eastern North Atlantic, studies have shown that there can be strong biogeographical shifts in copepod assemblages resulting from a northward extension of warm-water species and a decrease in colder-water species [3]–[4]. In the Northwest Atlantic including the Northeast U.S. continental shelf (NEUS), the abundance of small copepods including *Centropages typicus*, increased from 1990–2000, but the abundance of large copepods including *Calanus finmarchicus* remained constant or declined slightly during the same period [5]–[9]. The shifts in plankton are attributed to the relatively low salinity water that formed near the Canadian Archipelago during 1989 and propagated from the Labrador Sea to the NEUS [6]–[7].

The NEUS includes four major subareas: the Gulf of Maine (GOM), Georges Bank (GB), Southern New England (SNE) and the estuarine-dominated waters of the Mid-Atlantic Bight (MAB). The northern part of NEUS, including the GOM and GB, is affected by changes in the Arctic and subarctic region, whereas the MAB, the more southerly part of the NEUS ecosystem, is affected by complex water movements, including freshwater input, Gulf Stream influences and along-shelf advection from the north. In the MAB, zooplankton are comprised of species associated with different water masses [10]–[11]. The mean flow over the continental shelf and slope within the MAB is toward the southwest along the isobaths and this flow is stronger during winter and is weaker or reverses during summer [12]–[14]. There is strong evidence that the slope currents and plankton in the MAB are influenced by the Labrador Current [11], [15] and the Gulf Stream [13], [16]–[17].

Climate change likely has profound impacts on marine ecosystems and two different types of change in lower trophic levels are likely to have impacts on higher trophic levels: long term changes in species composition [18] and changes in phenology including demographic structure and seasonal patterns [19]–[20]. Zooplankton in the NEUS include Arctic-Boreal species, tropical-subtropical species, and many temperate species [10]–[11]. Temporal changes in zooplankton have been examined in several studies [7], [9], [11], [21]–[22], and these changes have been related to hydrographic variables, but have not been related to physical forcing explicitly. Meanwhile, knowledge of long term changes in abundance and seasonal patterns is critical for understanding how large scale ocean variability affects zooplankton in different regions.

To achieve a clearer understanding of the mechanistic linkage between large-scale forcing and plankton dynamics and the associated spatial scales, the present study focuses on two calanoid copepod species: the subarctic species *C. finmarchicus* and temperate species *Cen. typicus* [4]. For *C. finmarchicus*, we hypothesize that there will be no difference in decadal changes in abundance and seasonal peak time between the north (the GOM and GB) and south (the MAB). We predict that this hypothesis will be falsified and data will show that *C. finmarchicus* peaks in the north and declines in the south. When significant southerly transport is detected, *C. finmarchicus* abundances will increase. We predict that this will be shown by a significant, negative relationship between *C. finmarchicus,* southward alongshore transport and the GSNWI. For *Cen. typicus*, we hypothesize that there will be no difference in decadal changes in abundance and seasonal peak time between the north and south. We predict that this hypothesis will be falsified and data will show that data will show that *Cen. typicus* peaks in the south and declines in the north. When significant northward transport is detected, *Cen. typicus* abundances will increase. We predict that this will be shown by a significant, positive correlation between *Cen. typicus*, northward alongshore transport and the GSNWI.

## Data and Analysis

### 1 Ethics Statement

Shelf-wide plankton surveys are conducted 6–7 times per year over the continental shelf from Cape Hatteras, North Carolina to Cape Sable, Nova Scotia, by the Northeast Fisheries Science Center, the National Marine Fisheries Service. Zooplankton data are available at ftp://ftp.nefsc.noaa.gov/pub/dropoff/jhare/EcoMon_Data/and temperature data are available at http://www.nefsc.noaa.gov/epd/ocean/MainPage/ioos.html.

### 2 Zooplankton Data

Plankton samples were collected from 1977 to 2010 with a 61-cm bongo frame fitted with a net of 333 *µ*m mesh, towed obliquely to a maximum depth of 200 m or 5 m from the bottom and back to the surface with a flowmeter in the center of the Bongo frame to measure the volume of water filtered during the tow. Samples were preserved in 5% formalin and were sorted, counted, and identified to the lowest possible taxa. See Kane [7] for detailed information on survey cruises. Temperature profiles were measured with a CTD at each station and the mean temperature for the upper 3 m was used in this analysis. Because survey cruises did not cover the region at the same time each year, bi-monthly abundance was calculated with data from complete surveys whose midpoint fell within the two-month bin.

The present study focused on *C. finmarchicus* and *Cen. typicus*, which were among the most common zooplankton species and made up to ∼40% (16%–68%) of the total abundance. Data were binned to every two degrees, e.g., 35–36°N, to examine latitudinal variation in seasonal patterns. Time series analysis was performed in four sub-regions: the GOM, GB, SNE, and MAB, to investigate variation in seasonal pattern and long term abundance for the two species.

### 3 Time Series Analysis of the Long Term Trend

To examine the long term trend of two copepod species, we performed time series analysis. The principal of time series models is similar to regression model in which the explanatory variables are functions of time and the parameters are time-varying [23]. The simple univariate time-series models are based on a decomposition of the series into a number of components including a local trend, a deterministic seasonal component, and error terms,
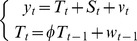
where 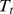 is the trend, 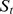 is the seasonal component, 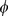 is the auto-correlation coefficient for 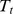, 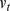 and 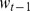 are independent error terms and both approximate a standard normal distribution 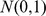. The coefficients were estimated using the Kalman Filter algorithm in Matrix Laboratory (MATLAB, [24]). The local trend (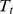) is autocorrelated, i.e., the trend at time 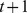 is a function of the trend at time 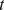. The seasonal component (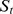) is modeled by the trigonometric seasonal model; a combination of sine and cosine functions with a seasonal period of 12 months [25].

The model was fit to zooplankton abundances from 1977 to 2009 at 6 bi-monthly points per year (n = 198). This allows for an estimate of long term trends and seasonal components: long term trends should be relatively smooth and the seasonal component should remain similar among years because the trigonometric seasonal model uses a combination of sine and cosine functions to model the recurrent patterns in the dataset. Samples were not collected in 1989–1991 in the south, therefore time series analyses were performed for two different time periods: 1977–1988 and 1992–2009.

Long term abundance, seasonal peak time and abundance were compared among four sub-regions. The observed data often had missing values which make it difficult to compare seasonal patterns, therefore we used fitted data from the univariate time series models. Note that the long term trends and seasonal changes estimated from the model indicate the relative changes in abundance rather than the absolute values, i.e., seasonal changes are relative to long term abundance, therefore the estimated seasonal changes are often negative.

### 4 Sea Level Anomaly and Geostrophic Current

Satellite altimetry can be an effective tool to study sea level variability over continental shelves, particularly for interannual, seasonal, and intra-annual variations (periods from 20 days to 1 year) [26]. These data have been used to estimate alongshore coastal current on the west coast [1], [27]–[28] and east coast [29]–[31] of the United States. Feng and Vandemark [30] reported the root mean square error of 3–4 cm s^−1^ and 6 cm s^−1^ between altimetry measurements and tide gauge and sea surface layer current measurement at the 60 day time scale for the MAB.

**Figure 1:**
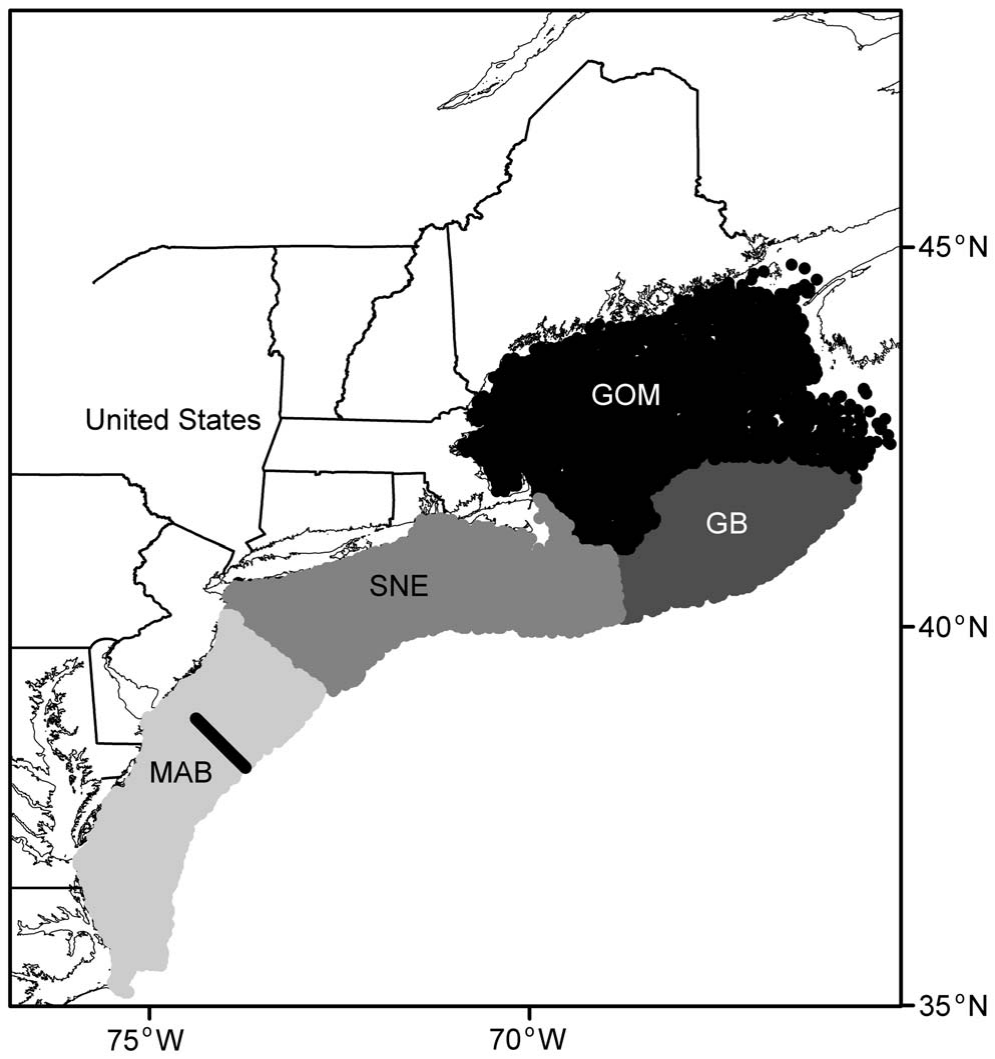
Zooplankton sampling area in the Northeast U.S. continental shelf including the Gulf of Maine (GOM), Georges Bank (GB), Southern New England (SNE) and the estuarine-dominated waters of the Mid-Atlantic Bight (MAB). The defined path for alongshore current derived from altimeter sea level data (black bar) at 39.3°N.

To analyze alongshore current velocities, gridded satellite altimeter sea level anomalies and geostrophic velocities were downloaded from AVISO (http://www.aviso.oceanobs.com/duacs/). We chose the delayed time, updated version of AVISO geostrophic velocities, gridded weekly at 1/3° in a rectangular projection for 35°N –45°N from October 14, 1992– September 28, 2011. In the present study, we calculated alongshore current velocities at 39°N within 2° of the coast. To avoid land contamination, we discarded data from the first two gridded cells and calculated a mean alongshore current velocity for the remaining 4 gridded cells (Figure 1). To remove high-frequency signals and match the zooplankton data, bi-monthly average velocities and anomalies were calculated. Annual cumulative northward current (positive values) and southward current (negative values) were calculated to examine interannual variation.

**Figure 2:**
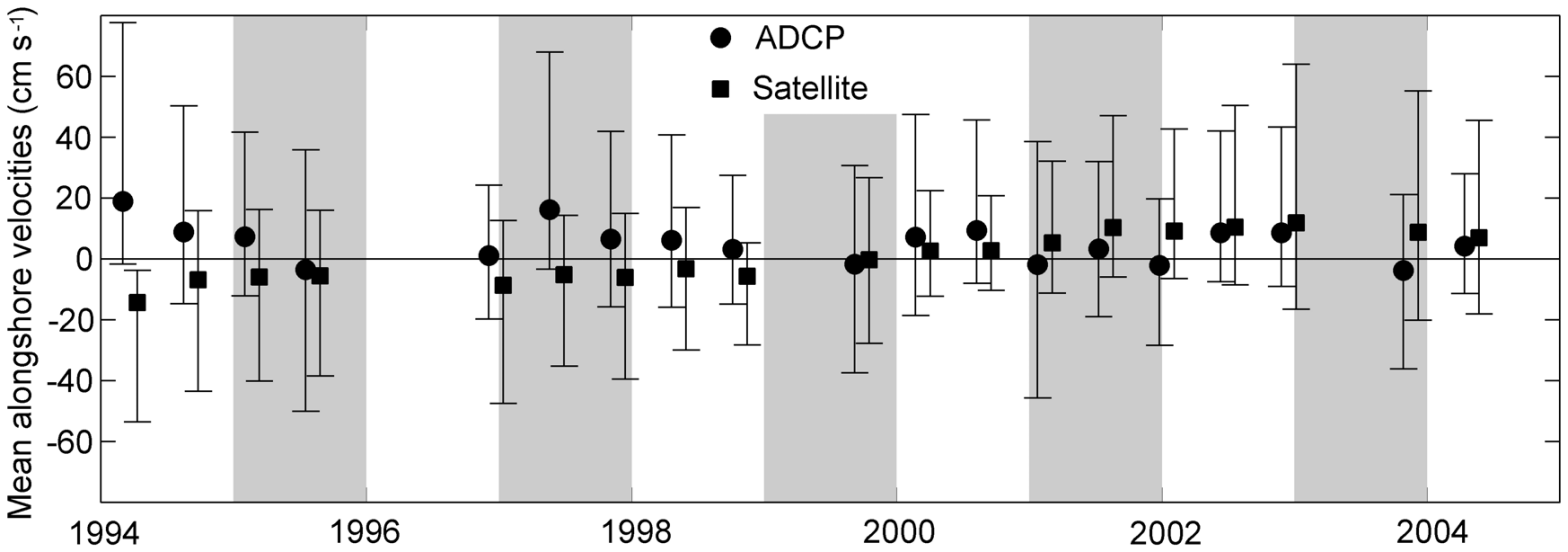
Comparison of satellite altimetry derived alongshore current and measurements from Acoustic Doppler Current Profiler along a spatially close track line. Shaded bars represent alternate years.

The problem of applicability of satellite altimetry data to the NEUS continental shelf is of interest because the shelf tends to be relatively shallow and affected by tides. Hence, it is important to verify the estimates of geostrophic current from satellite altimetry data. We downloaded hourly Acoustic Doppler Current Profiler (ADCP) measurements for the upper 100 m between 39°N –40°N within 2° of the coastline from the Oleander Project (http://po.msrc.sunysb.edu/Oleander/). For details, see Flagg *et al.* [16], [32]. The hourly ADCP measurements were first binned into weekly data to match the satellite altimetry data for each location. Then, the estimate at each point was compared to the satellite estimate from the same grid. Because satellite estimates were at a weekly time scale and 1/3° spatial grid and ADCP estimates were at an hourly scale in most cases at a single spatial point, it was difficult to formulate a rigorous statistical comparison. When comparing the satellite derived alongshore current velocities to the ADCP estimates in the same area and time frame, in general satellite derived values were consistent with ADCP estimates, except that ADCP measurements tended to have more northward measurements and show larger variation in the 1990s (Figure 2). Furthermore, Lillibridge and Mariano [33] showed that alongtrack altimetry data were consistent with the ADCP measurements from the Oleander Project.

### 5 The Gulf Stream North Wall Index (GSNWI)

The GSNWI was constructed from the latitude of the north wall at each of the six longitudes (79, 75, 72, 70, 67 and 65°W) through principal component analysis [34]. The index was calculated as a weighted average of the standardized latitude series where the weighting factors are derived from principal component analysis and it has positive correlation coefficients of about 0.5 with the latitude of the north wall at each of the six longitudes. The details and full data are available at http://www.pml-gulfstream.org.uk/data.htm.

## Results

### 1 Spatial and Seasonal Patterns

**Figure 3:**
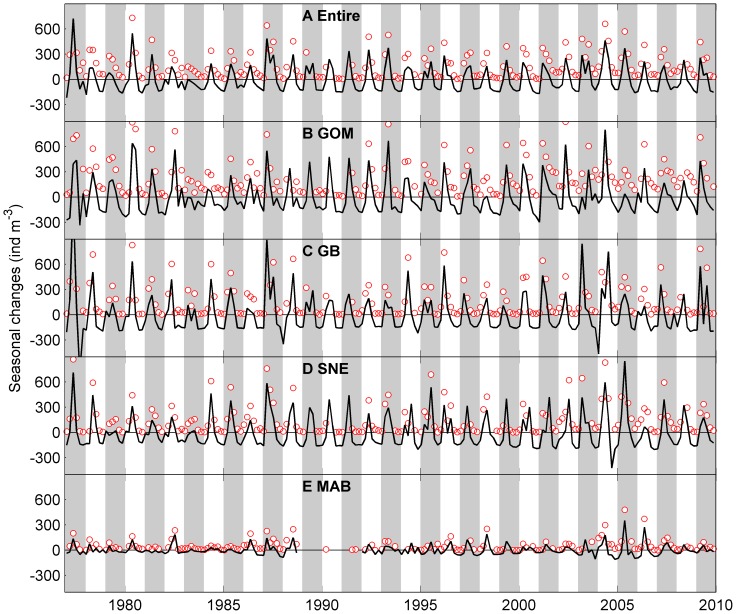
Bimonthly mean abundance of *Calanus finmarchicus*, a large subarctic associated copepod in the study area. A: entire region, B: Gulf of Maine (GOM), C: Georges Bank (GB), D: Southern New England (SNE) and E: Mid-Atlantic Bight (MAB). Red circles represent observed bi-monthly abundance. Solid black lines represent seasonal patterns determined from the univariate time series analysis, which indicate relative changes to the long term trend rather than absolute abundances. Shaded bars represent alternate years.

**Figure 4:**
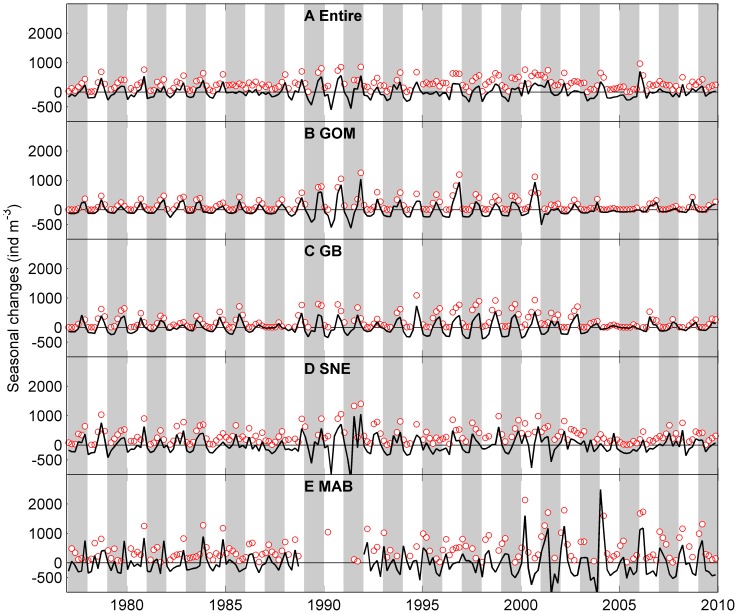
Bimonthly mean abundance of *Centropages typicus* a small temperate coastal copepod in the study area. A: entire region, B: Gulf of Maine (GOM), C: Georges Bank (GB), D: Southern New England (SNE) and E: Mid-Atlantic Bight (MAB). Red circles represent observed bi-monthly abundance. Solid black lines represent seasonal patterns determined from the univariate time series analysis, which indicate relative changes to the long term trend rather than absolute abundances. Shaded bars represent alternate years.

*C. finmarchicus* and *Cen. typicus* exhibited different spatial and temporal patterns (Figures 3 and 4). *C. finmarchicus* had the highest abundance in the GOM (233±213 ind m^−3^, mean ± standard deviation) and GB (183±243 ind m^−3^), followed by the SNE (152±196 ind m^−3^), and the lowest mean abundance in the MAB (55±73 ind m^−3^). *Cen. typicus* showed the opposite spatial patterns with the highest mean abundance in the MAB (493±468 ind m^−3^), followed by the SNE (342±276 ind m^−3^), and lowest abundance in the GB (211±256 ind m^−3^) and GOM (169±243 ind m^−3^).

**Figure 5:**
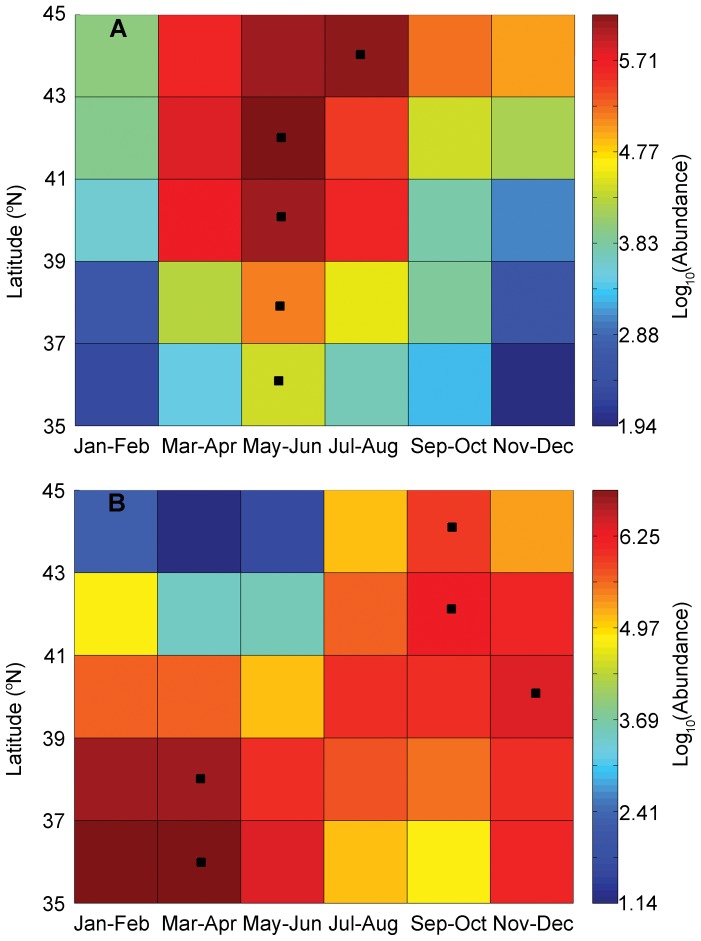
Latitudinal variation in seasonal patterns. Bi-monthly log transformed mean abundance of *Calanus finmarchicus* (A) and *Centropages typicus* (B) from 1977–2009 within each region (binned by 2° latitudes) of the US northeast shelf ecosystem. Numbers are individual m^−3^. Warm color (red) represents high abundance and cold color (blue) represents low abundance. Black squares indicate the peak month.

*C. finmarchicus* showed a single annual peak in May – June throughout the entire study region except in the most northern area (43–44°N) where the peak time extended to July- August (Figures 5A). *Cen. typicus* also had a single annual peak and relatively large variation between north and south (Figure 5B). The peak time mostly occurred in September – December in the north, but January – April in the south with a second peak in November - December.

### 2 Long Term Variation in Seasonal Abundance

**Figure 6:**
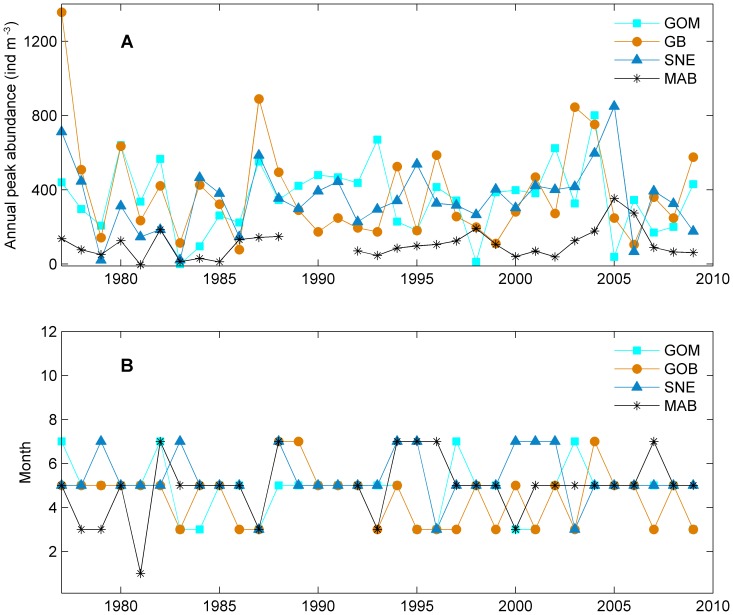
Annual peak abundance and time of *Calanus finmarchicus* in the Gulf of Maine (GOM), Georges Bank (GB), Southern New England (SNE), and Mid-Atlantic Bight (MAB). A: peak abundance and B: peak time.

The seasonal patterns derived from the univariate time series analysis suggested that the *C. finmarchicus* seasonal peak abundances had relatively large interannual variation in the SNE, GOM and GB (Figure 3 and Figure 6A). The peak abundance in the MAB tended to be relatively low with less interannual variation compared to other sub-regions. Note that the peak abundance declined after 2005 in all four sub-regions. The seasonal peak time remained consistent throughout the study period in all four regions: most peaks were in May – June with a few years in March – April and July – August (Figure 3 and Figure 6B).

**Figure 7:**
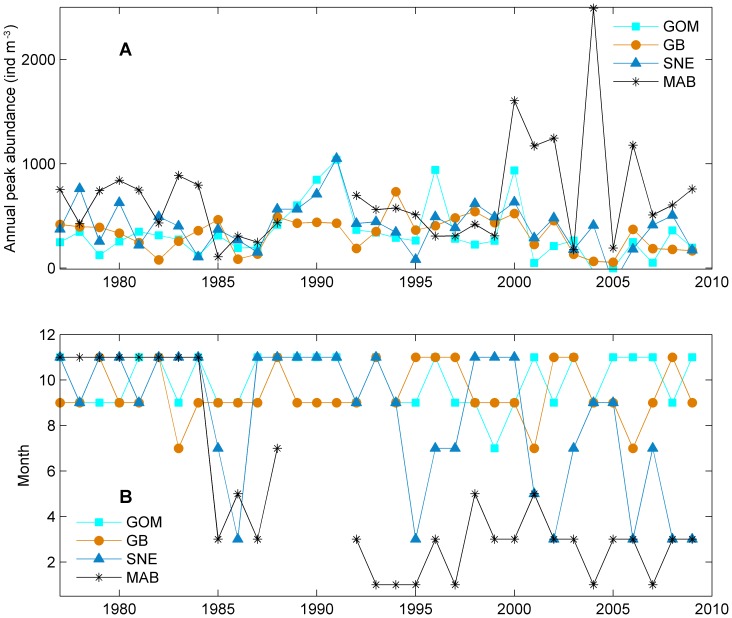
Annual peak abundance and time of *Centropages typicus* in the Gulf of Maine (GOM), Georges Bank (GB), Southern New England (SNE), and Mid-Atlantic Bight (MAB). A: peak abundance and B: peak time.

*Cen. typicus* seasonal peak abundances were more consistent in three sub-regions before 2000 (Figure 4 and Figure 7A). In 2000–2005, the seasonal peak abundance in the MAB was much higher than other sub-regions. However, seasonal peak time showed large spatial and temporal variation (Figure 7B). In the MAB, the peak time switched from November – December to January – February or March – April after 1985 except in 1988, 1998 and 2001 when the peak time was in May – June. In the SNE, the peak time switch was more variable, but mostly in November – December before 1995 except 1985–1986. After 1995, the seasonal peak time in the SNE only occurred in November – December in 1998–2000 and fluctuated between March – April and September - October in other years. In the GOM and GB, the peak time was mostly in November – December.

### 3 Spatial Variation in Long Term Changes

**Figure 8:**
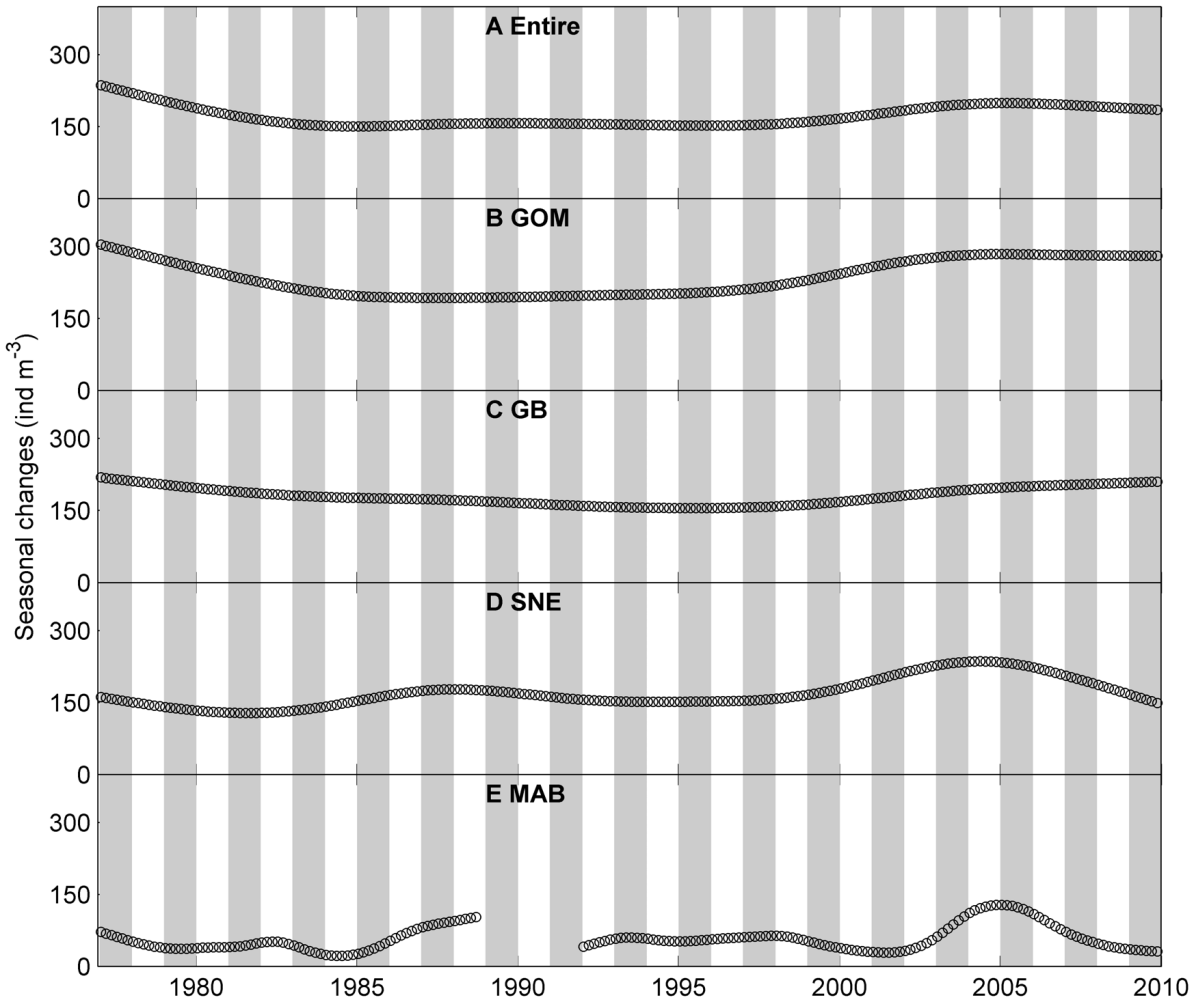
Long term changes of *Calanus finmarchicus* in the study area. A: entire region, B: Gulf of Maine (GOM), C: Georges Bank (GB), D: Southern New England (SNE) and E: Mid-Atlantic Bight (MAB). The long term trend was estimated from time series of bi-monthly mean abundance in each subregion by removing seasonal variation. Shaded bars represent alternate years.

**Figure 9:**
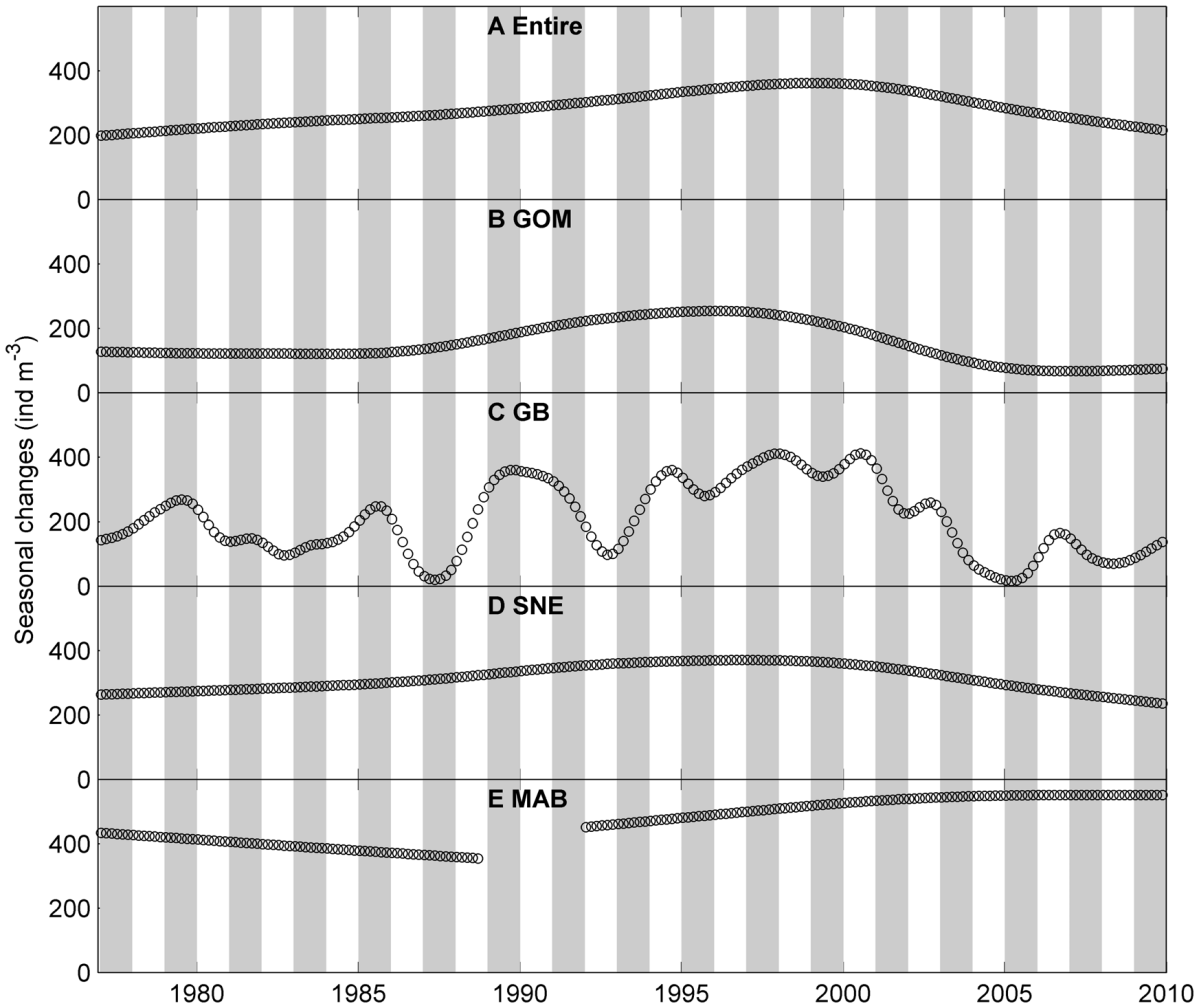
Long term changes of *Centropages typicus* in the study area. A: entire region, B: Gulf of Maine (GOM), C: Georges Bank (GB), D: Southern New England (SNE) and E: Mid-Atlantic Bight (MAB). The long term trend was estimated from time series of bi-monthly mean abundance in each subregion by removing seasonal variation. Shaded bars represent alternate years.

The long term trends derived from the univariate time series analysis showed much less interannual variation than seasonal variation (Figures 8 and 9). The long term variation of *C. finmarchicus* in the entire region ranged from ∼150–236 ind m^−3^, whereas the seasonal variation ranged from −217–724 ind m^−3^. The long term variation of *Cen. typicus* ranged from 198–362 ind m^−3^, whereas the seasonal variation ranged from −603–700 ind m^−3^.

The long term variation of *C. finmarchicus* in the entire region declined from 1977–1980 and was followed by two periods with elevated abundance: 1984–1990 and 2000–2006 (Figure 8). In the GOM and GB, the long term variation of *C. finmarchicus* was consistent with the overall trend and showed a relatively small range from 193–304 ind m^−3^ in the GOM and 155–219 ind m^−3^ in the GB. The long term trend of *C. finmarchicus* showed more variation in the SNE (128–236 ind m^−3^), but less variation than the MAB (22–128 ind m^−3^). *C. finmarchicus* showed slightly elevated abundance in 1985–1990 and 2000–2005 in the MAB and SNE, i.e., the long term trend of *C. finmarchicus* was similar in the MAB and SNE.

The long term variation of *Cen. typicus* gradually increased in 1977–2000 and declined afterwards (Figure 9). The long term variation in the SNE and GOM were similar, both were consistent with the overall long term trend. However, the long term pattern was remarkably different in the MAB compared to the GB. In the MAB, the long term variation declined from 1977–1990 and increased after 1992. In the GB, *Cen. typicus* had an elevated abundance in 1987–2001 and showed a relatively larger fluctuation than other sub-regions.

### 4 Alongshore Transport, the GSNWI and Local Temperature and Copepods

**Figure 10:**
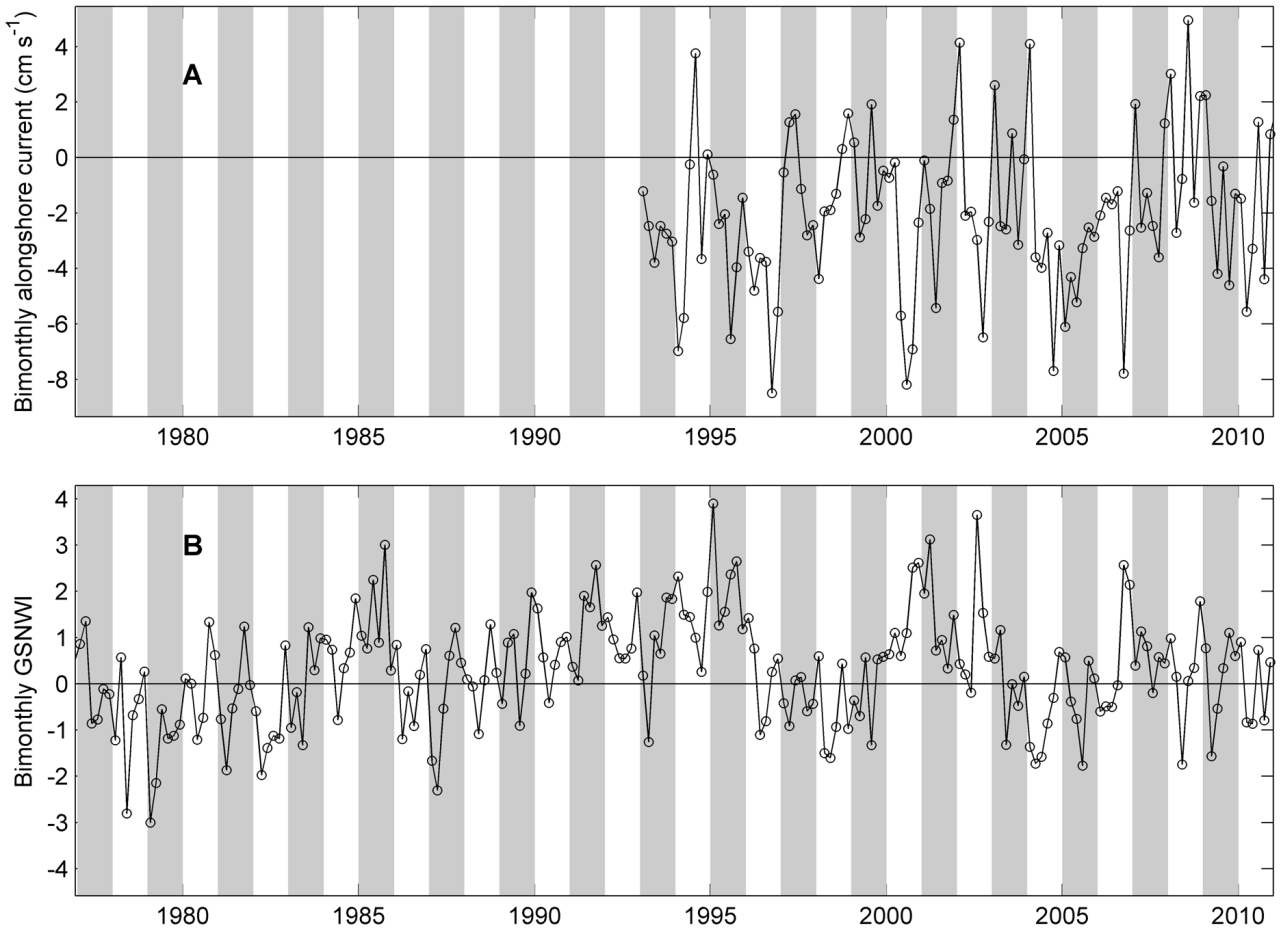
Bimonthly alongshore current velocities the Gulf Stream North Wall Index (GSNWI). A: Bi-monthly alongshore current velocities with positive values indicating northward current and negative current indicating southward current. B: Bi-monthly values of GSNWI. Shaded bars represent alternate years.

The bi-monthly satellite derived mean alongshore current over the MAB shelf estimated from satellite altimetry data was mostly southward but with large inter- and intra- annual variations (Figure 10A). The strong northward or weakened southward current was clear in 1996–1999 and 2005–2010. The peak abundance of *C. finmarchicus* in the entire region showed negative relationship with the alongshore current, i.e., strong southward water movement relates to higher peak abundance (Table S1 in File S1). The negative relationship between the alongshore transport and *C. finmarchicus* peak abundance persisted in all four sub-regions, but only significantly in the SNE. The peak abundance of *Cen. typicus* did not show significant relationship with the alongshore transport (Table S1 in File S1).

The bi-monthly GSNWI varied from −3 to 3.9 during the study period with mostly positive anomalies in 1985–1995 and 2000–2004 (Figure 10B) which appeared to be coincident with the elevated level of long term abundance for *Cen. typicus* in the GB. The peak abundance of *C. finmarchicus* only showed significant correlation with the GSNWI in the MAB (Table S2 in File S1), whereas the peak abundance of *Cen. typicus* showed significant correlation with the GSNWI in the GOM and GB. Furthermore, local temperature did not show significant correlations with the abundance of *C. finmarchicus* and *Cen. typicus* in any of the four sub-regions (Table S3 in File S1).

**Figure 11:**
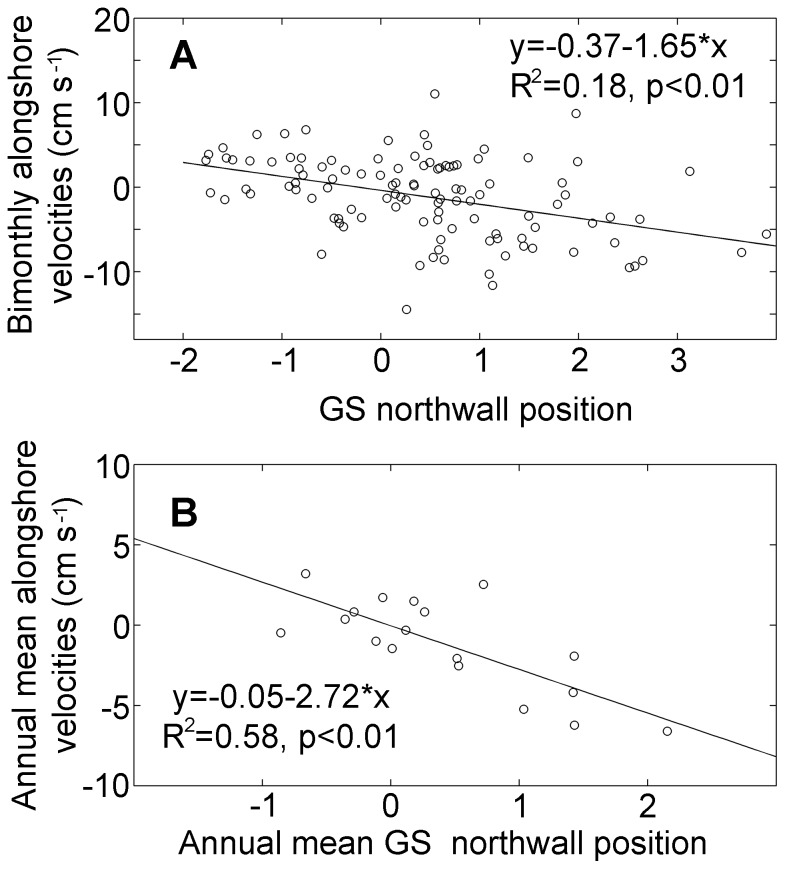
Regression analysis for alongshore velocities and the Gulf Stream north wall index (GSNWI). A: bi-monthly alongshore velocities from satellite altimetry data and GSNWI and B: annual mean velocities and mean GSNWI.

The GSNWI was negatively correlated with alongshore transport (Figure 11), i.e., when the Gulf Stream north wall was southward, the southward alongshore transport was strong. The negative relationship between the GSNWI and alongshore velocities persisted at both bimonthly (Figure 11A) and annual scales (Figure 11B). However the relationship is quite variable depending on the location of the defined pathway for transport.

## Discussion

### 1 Seasonal Patterns

The current study highlights the importance of the spatial and temporal scales in understanding seasonal dynamics of marine populations. For example, we showed that the timing of the seasonal peak for *Cen. typicus* shifted from September – December to January - February in the MAB after 1985 and in the SNE the seasonal shift was more variable. Durbin and Kane [21] investigated seasonal patterns for *Cen. typicus* using the dataset (1977–2003) and concluded that *Cen. typicus* in the MAB did not show strong seasonal variation with two peaks in January – March and September – December. The present study showed that *Cen. typicus* only showed one seasonal peak, the second peak (September – December) in the previous study was actually caused by a shift in the timing of the seasonal peak. The shift was also observed in the SNE, especially in late the late 1990s and 2000s. The occurrence of the shift in the MAB and SNE suggests that the NEUS system underwent significant changes, but the potential causes for the shift in annual peak time remain elusive.

The univariate time series analysis suggested that seasonal abundance also showed large interannual variation and it explained a large portion of the interannual variation for both species. Therefore, knowledge of population processes and related local physical and biological processes is critical to understand the interannual variation of copepod abundance in the NEUS. For example, Ji *et al.* [35] found that the abundance of *Cen. typicus* was significantly correlated with predator abundance in the GB. Liu *et al*. [36] reported that significant dynamical interaction and coherence between *Cen. typicus* and Atlantic mackerel and haddock on Georges Bank. Other factors affecting fecundity and growth, such as food quality and availability and local temperature, need to be considered to understand the seasonal dynamics and the interannual variation in seasonal dynamics.

The shift in seasonal peak time has important ramifications for trophic interactions, altering food web structure, and eventual ecosystem level changes [19]. In the south, many fish larvae rely on zooplankton [37]. For example, Atlantic menhaden (*Brevoortia tyrannus*) larvae generally peaked in September – October in the MAB and they chiefly feed on small copepods such as *Cen. typicus* [38]–[39]. The potential mismatch in the timing of the seasonal peak between Atlantic menhaden larvae and *Cen. typicus* may have impacts on early recruitment.

### 2 Spatial Distribution and Long Term Changes

The abundance of two copepod species showed large north-south variability and the comparative approach provides a useful tool to investigate the structure and scales in the NEUS. *C. finmarchicus*, a subarctic species, was more abundant in the GOM and GB, but less abundant in the MAB. In contrast, *Cen. typicus* was more abundant in the MAB, but less abundant in the GOM and GB. Meanwhile, the SNE appeared to be a mixed region because the abundance of *C. finmarchicus* was generally higher than in the south, and the abundance of *Cen. typicus* was generally higher than in the north. The distinct spatial structure offered a unique opportunity to understand the dynamics of two copepod species and their potential drivers.

The long term trends in four sub-regions showed large differences, particularly between the MAB and GB. The long term trends across the entire region for *C. finmarchicus* were relatively stable and consistent with the trends in the GOM and GB. But the long term trends of *C. finmarchicus* were more variable in the MAB, whereas the long term trends of *Cen. typicus* tended to be more variable in the GB. This pattern is consistent with the general spatial distribution pattern: if we consider that *C. finmarchicus*, a subarctic species, is advected to the south (MAB) from the subarctic waters of the North Atlantic, and *Cen. typicus* as a temperate coastal species propagating from south to north, it is reasonable to expect changes would be more obvious for *C. finmarchicus* in the south and *Cen. typicus* in the north. Hare and Kane [40] emphasized that the historical context, i.e., temporal scale, is important in understanding the observed copepod abundance data. The present study highlights that the observed changes need to be considered in the proper spatial context. For example, the observed long term changes for *Cen. typicus* in the north can not be extended to the south, and the observed long term changes for *C. finmarchicus* in the south can not be extended to the north because the long term changes were different between the north and south for both species.

The long term changes in the four sub-regions were different for both species, but the drivers remains elusive. The present study extends previous studies of zooplankton from the Northwest Atlantic shelf, in which many different large scale forcings were related to different copepod species. Low salinity water from the Labrador Sea could affect copepod abundance in the Northwest Atlantic, specifically the increasing abundance of small copepods [6], [8], [41]. The abundance of *C. finmarchicus* in the NEUS has been related to the North Atlantic Oscillation (NAO) index [42]. Furthermore, Kane [22] examined the copepod assemblage and species abundance in relation to the Atlantic Multidecadal Oscillation (AMO). Biological mechanisms, such as top-down control, i.e., predation, have been considered as factors that could affect the abundance of *Cen. typicus* in the GB, but not in the GOM [35]. Other factors such as food quality and availability and local temperature can also affect long term trend through regulating population growth and fecundity.

The present study considers transport as a mechanistic linkage between large scale forcing and local ecosystem structure. Most large scale indicators, e.g., NAO and AMO, reflect changes in atmosphere or sea surface temperature at regional and basin scales, and do not directly reflect changes in the water column. Furthermore, changes in the Labrador Sea reflect changes in a remote location. Zooplankton are by definition drifters, therefore it is reasonable to consider alongshore transport as a mechanism linking large scale forcing and local zooplankton dynamics. The present study provides evidence that alongshore transport could serve as the linkage between large scale forcing (e.g., the Gulf Stream), and lower trophic levels. For example, the abundance *C. finmarchicus* was negatively correlated with alongshore transport, i.e., when alongshore transport was negative, more water flowing from the north, the abundance of *C. finmarchicus* was high. The abundance of *Cen. typicus* did not show a significant correlation with alongshore transport, but was positively correlated with the GSNWI which is a measure of the transport of the Gulf Stream system [43] from south. Furthermore, alongshore transport was negatively correlated with the GSNWI. Overall, results suggested that the abundance of *C. finmarchicus*, a subarctic species, was high when there was more water coming from the north and the abundance of *Cen. typicus*, a temperate coastal species was positively related to the GSNWI. One of the potential issues with this study is the relatively low correlation between the abundance of *C. finmarchicus* and alongshore transport and the abundance of *Cen. typicus* and the GSNWI. One of the reasons might be that the NEUS is a complicated system and it is difficult to estimate alongshore transport representing the entire region from a fixed location. At the same time, the resolution of satellite altimetry data was insufficient to characterize alongshore transport at the same spatial scale as our zooplankton samples. Another potential consideration is the intrinsic dynamics of marine organisms. A recent study found that copepod species exhibit clear nonlinearity on Georges Bank [36]. In nonlinear systems, forcing and response variables may show weak or no correlation despite clear deterministic, mechanistic relationships among them. Therefore, correlation-based approaches may be not sufficient in such a case.

### 3 Large Scale Forcing and other Factors

The northwest Atlantic slope waters respond as a coupled system to major changes in climate [44]–[45]. The strength of the Labrador Current is inconsistent with the NAO index: the Labrador Current is stronger when the NAO is positive [46]–[47], but Rossby and Benway [14] reported an opposite relationship: when the NAO is positive, the Labrador Current is weaker. Fluctuations in the NAO index are correlated with the location of the Gulf Stream [34], [48] whereby high values of the NAO index correspond to more northerly paths of the Stream. Furthermore, there have been changes in outflow of low salinity water from the Arctic and a general freshening of shelf waters from the Labrador Sea to the MAB beginning in the late 1980s [6], [49]. This freshening has altered circulation and stratification patterns and has been linked to changes in the abundances and seasonal cycles of phytoplankton, zooplankton, and fish [6], [18]. Meanwhile, other inputs such as St. Lawrence discharge can affect Scotian shelf flow by increasing outflow of relatively fresh surface water from the gulf to the eastern Scotian shelf and penetration of slope water at depth onto the shelves [50].

To understand the relationship between long term changes in copepod abundance and large scale forcing, it is necessary to identify local factors that can manifest the changes in large scale forcing. For example, Hare and Kane [40] proposed a conceptual model to examine the abundance of *C. finmarchicus* in the GOM in relation to large scale variability. As the NAO fluctuates, the circulation patterns in the northwest Atlantic vary and the coupled slope water system is affected, as indexed by the regional slope water temperature, which in turn affects *C. finmarchicus*. Local sea surface temperature also affects *C. finmarchicus*; potentially through physiological processes (e.g., metabolism) and population processes (e.g., growth, recruitment) or indirectly through trophic interactions and advective influences. The present study indicated both copepod species showed different long term trends between north and south. To understand the causes for the observed difference between north and south, it is necessary to identify local factors that can reflect how different regions respond to large scale variability.

The present study sheds light on the linkage between copepods and ocean transport, which could facilitate our understanding on ecosystem dynamics. However, there are several observations that would benefit from more thorough investigation in the future. First, the long term change of *Cen. typicus* in the north is puzzling: the decline after 2000 could not be explained by salinity because the salinity tended to be low in the north [38]. Second, the annual peak time for *Cen. typicus* switched from November – December to January - February in the south (after 1985) and central regions (after 2000). Third, from the spatial distribution patterns of two copepod species, it appeared that *C. finmarchicus* can be transported from the GOM and GB to the SNE, and *Cen. typicus* can be transported from the MAB to the SNE, but the low abundance of *C. finmarchicus* in the south and the low abundance of *Cen. typicus* in the north suggest that they may experience high advection loss or high mortality in the middle region.

Although over broad time and space scales, the dynamics of marine zooplankton is controlled by bottom-up rather than top-down processes, the intrinsic biological processes and trophic interactions among adjacent trophic levels become prominent at smaller spatial and shorter temporal scales [51]–[52]. For example, Liu et al [36] found that *C. finmarchicus* tend to exhibit nonlinear dynamics, but *Cen.typicus* does not in the GB eco-region. Moreover, *C. finmarchicus* showed strong dynamical interaction and coherence with Atlantic herring, while *Cen. typicus* tend to dynamically interact and associate with Atlantic markerel and haddock on Georges Bank. Given the complex dynamics of marine organisms, toward a thorough understanding of these questions, we need to synergistically consider both physical forcing i.e., cross-shelf advection and local hydrographic conditions, and ecological processes i.e., food quality and concentration, and detailed information on population processes in different regions.

## Supporting Information

File S1 **This file contains Table S1–Table S3.** Table S1, Regression analysis between copepod abundance and alongshore transport. Table S2, Copepod abundance and the Gulf Stream north wall index. Table S3, copepod abundance and local temperature (in four different sub-regions: the Gulf of Maine (GOM), Georges Bank (GB), Southern New English (SNE) and Middle Atlantic Bight (MAB).
(DOCX)
