# Supplementary material for: Decadal Changes in Zooplankton of the Northeast U.S. Continental Shelf
Source: PLoS One. 2014 Jan 31;9(1):e87720. doi: 10.1371/journal.pone.0087720 (PMC3909209; doi:10.1371/journal.pone.0087720)
Supplement: File S1 — This file contains Table S1–Table S3. Table S1, Regression analysis between copepod abundance and alongshore transport. Table S2, Copepod abundance and the Gulf Stream north wall index. Table S3, copepod abundance and local temperature (in four different sub-regions: the Gulf of Maine (GOM), Georges Bank (GB), Southern New English (SNE) and Middle Atlantic Bight (MAB). (DOCX) [file pone.0087720.s001.docx]

Table S1 Regression analysis between copepod abundance and alongshore transport in four different sub-regions: the Gulf of Maine (GOM), Georges Bank (GB), Southern New English (SNE) and Middle Atlantic Bight (MAB). The predictor variable is annual mean alongshore velocities and the response variable is the log-transformed peak abundance for two copepod species.

| Region | *Calanus finmarchicus* | *Centropages typicus* |
| --- | --- | --- |
| Entire region | $y=5.73-0.12*x$  *R^2^* = 0.35, *p* < 0.01 | $y=6.25-0.01*x$  *R^2^* = 0.01, *p* = 0.88 |
| GOM | $y=6.16-0.07*x$  *R^2^* = 0.06, *p* = 0.35 | $y=5.74-0.04*x$  *R^2^* = 0.01, *p* = 0.80 |
| GB | $y=6.01-0.08*x$  *R^2^* = 0.07, *p* = 0.32 | $y=6.11-0.01*x$  *R^2^* = 0.01, *p* = 0.88 |
| SNE | $y=5.49-0.21*x$  *R^2^* = 0.20, *p* =0.05 | $y=6.58+0.09*x$  *R^2^* = 0.07, *p* = 0.29 |
| MAB | $y=4.04-0.28*x$  *R^2^* = 0.18, *p* = 0.07 | $y=6.95-0.04*x$  *R^2^* = 0.02, *p* = 0.60 |

Table S2 Regression analysis between copepod abundance and the Gulf Stream north wall index (GSNWI) in four different sub-regions: the Gulf of Maine (GOM), Georges Bank (GB), Southern New English (SNE) and Middle Atlantic Bight (MAB). The predictor variable is annual mean GSNWI and the response variable is the log-transformed peak abundance for two copepod species.

| Region | *Calanus finmarchicus* | *Centropages typicus* |
| --- | --- | --- |
| Entire region | $y=5.95-0.08*x$  *R^2^* = 0.02, *p* = 0.38 | $y=6.26+0.07*x$  *R^2^* = 0.03, *p* = 0.37 |
| GOM | $y=6.15-0.10*x$  *R^2^* = 0.02, *p* = 0.48 | $y=5.89+0.30*x$  *R^2^* = 0.12, *p* = 0.05 |
| GB | $y=6.20-0.14*x$  *R^2^* = 0.05, *p* = 0.22 | $y=5.99+0.32*x$  *R^2^* = 0.15, *p* = 0.02 |
| SNE | $y=5.78-0.07*x$  *R^2^* = 0.01, *p* = 0.73 | $y=6.46+0.10*x$  *R^2^* = 0.04, *p* = 0.28 |
| MAB | $y=4.64-0.55*x$  *R^2^* = 0.19, *p* = 0.01 | $y=6.79+0.02*x$  *R^2^* = 0.01, *p* = 0.86 |

Table S3 Regression analysis between copepod abundance and local temperature in four different sub-regions: the Gulf of Maine (GOM), Georges Bank (GB), Southern New English (SNE) and Middle Atlantic Bight (MAB). The predictor variable is annual mean temperature and the response variable is the log-transformed peak abundance for two copepod species.

| Region | *Calanus finmarchicus* | *Centropages typicus* |
| --- | --- | --- |
| Entire region | $y=4.07+0.08*x$  *R^2^* = 0.07, *p* = 0.14 | $y=5.59+0.01*x$  *R^2^* = 0.01, *p* = 0.92 |
| GOM | $y=4.81+0.05*x$  *R^2^* = 0.02, *p* = 0.30 | $y=5.46-0.04*x$  *R^2^* = 0.02, *p* = 0.61 |
| GB | $y=4.75+0.03*x$  *R^2^* = 0.05, *p* = 0.29 | $y=5.41-0.02*x$  *R^2^* = 0.01, *p* = 0.60 |
| SNE | $y=5.06+0.03*x$  *R^2^* = 0.01, *p* = 0.78 | $y=5.32+0.04*x$  *R^2^* = 0.02, *p* = 0.47 |
| MAB | $y=2.41+0.11*x$  *R^2^* = 0.03, *p* = 0.39 | $y=6.17-0.01*x$  *R^2^* = 0.01, *p* = 0.95 |
